# Explaining mobilization for revolts by private interests and kinship relations

**DOI:** 10.1177/10434631231219954

**Published:** 2023-12-06

**Authors:** Niccolò G. Armandola, Malte Doehne, Katja Rost

**Affiliations:** Department of Sociology, 27217 University of Zurich, Zurich, Switzerland

**Keywords:** Private interests, revolts, kinship relations, social networks, civil uprisings

## Abstract

Mobilization for revolts poses a significant challenge for rational choice theory because revolts are vulnerable to free-riding, which disincentivizes rational actors from mobilizing. Strong, informal relations such as kinship ties have been identified as factors that can shift the rational calculations of individuals and lead to mobilization for revolts. In social networks that are polarized by the presence of mobilized individuals, such as rebels, and actors opposing the mobilization effort such as the elite, kinship relations have not only a bridging effect but also a diverging one. Building on Tullock’s private interest theory, we develop a framework in which kinship relations determine the extent of individual’s payoffs and costs of mobilization for revolts against an elite. We posit that distant kin of the elite expect high payoffs of mobilization for revolts and face the lowest costs of mobilization for revolts by virtue of their position in the network of kinship relations. Using a unique, hand-collected dataset that reconstructs a revolt in Basel, Switzerland, in 1691, we test our framework and contribute to a better relational understanding of the mechanisms that lead rational actors to mobilize for revolts. Our analyses show that mobilization for revolts is mainly driven by distant kinship relations to the ruling elite rather than close kinship relations to the rebels.

## Introduction

Revolts are vulnerable to free-riding: as their success benefits large groups, individuals may choose not to participate, expecting others to bear the risks and costs associated with collective action (McAdam, 1986; Gould, 1993; Lu and Tao, 2017; Braun, 2018). This public-good aspect discourages individual mobilization, making it unappealing for rational actors to engage in revolts (Olson, 1965; Tullock, 1971; Opp, 2009, 2012; Sandler, 2015; Zhang and Yu, 2018; Shadmehr, 2018). Consequently, mobilization for revolts presents a significant challenge for rational choice scholars: why would rational actors engage in perilous mobilization efforts with low chances of success and only marginal individual benefits? What factors lead rational individuals to deviate from the optimal free-riding strategy and mobilize for revolts?

Tullock’s paradox of revolution (1971) addresses these questions with a private interest theory that shows how individual incentives play an important role in mobilization for revolts. He claims that the marginal contribution of an individual to the mobilization effort’s success is small and almost imperceptible to the actor. Thus, whether individuals are mobilized for revolts depends on their private interests rather than on their perceived possibility of contributing to the public good (Olson, 1965; Tullock, 1971; Silver, 1974). When mobilization for revolts is modeled as a private interest problem, questions arise about which factors impact the rational assessment of the payoffs and costs of mobilization (Opp, 2009, 2012; Zhou and Wang, 2018). Social relations, in particular informal interpersonal ties such as friendship and kinship relations (Della Porta, 1988; Gould, 1993; McAdam and Paulsen, 1993; Opp and Gern, 1993), have been identified as strong factors that can shift the rational calculations of individuals (Goldstone, 1994; Zhou and Wang, 2018).

Extensive research has studied the effect of informal social relations on mobilization against institutions such as the government and the state (for overviews, see Baldassarri, 2009; Buechler, 2016; Della Porta and Diani, 2020; McAdam, 2003; McAdam et al., 1996; Opp, 2009). In the absence of clear organizational structures, informal social ties become particularly efficient vehicles of mobilization (Gould, 1993): having close kinship relations to mobilized individuals increases the likelihood of being mobilized (Braun, 2018; Della Porta, 1988; Lu, 2019; Lu and Tao, 2017; McAdam, 1986; McAdam and Paulsen, 1993; Opp, 2012; Opp and Gern, 1993). However, the role of informal ties in settings where the target of the mobilization effort is a group of powerful actors rather than institutions remains understudied. Consequently, little is known about the effect of kinship relations to powerful actors such as ruling elites that oppose mobilization efforts. This is surprising, because a rational-choice framework conceives of both the oppressed and the oppressors as rational actors (Funcke and Franke, 2016; Olsson-Yaouzis, 2010; Tullock, 1971).

Our research addresses the question how social relations to both the elite and the rebels impact individuals’ mobilization for revolts against the elite. Building on Tullock’s (1971) private interest theory, we develop a framework in which kinship relations in social networks determine the extent of individuals’ payoffs and costs of mobilization for revolts. This framework considers four states of kinship relations: (1) close kin of the elite, (2) distant kin of the elite, (3) close kin of the rebels, and (4) distant kin of the rebels.^1^ We argue that in revolts, counterintuitively, distant kin of the elite are the most likely to mobilize for revolts, because their weak ties to the polarized network of elite members and rebels put them in a sweet spot that allows them to derive the highest payoffs from mobilization for revolts while facing comparatively low costs. Close kin of the rebels are expected to derive equally high payoffs from mobilization for revolts, but they face higher costs. These assumptions contrast with previous findings, which show that people are mobilized through close social relations to other mobilized actors.

We test our proposed framework on a well-documented historical case known as the *Einundneunziger Wesen*, a revolt that took place in 1691 in Basel, one of the largest city-states of the Swiss Confederacy in the early modern period (Röthlin 1986). Following tensions with neighboring France due to controversial decisions by the two mayors of Basel and corruption and nepotism allegations against the political elite, on March 24th, 1691, insurgent citizens besieged the city hall. They forced several politicians to resign and claimed control of the city’s parliament (Schweizer, 1931). However, the political elite regained control of the parliament and city a few months later and publicly executed three of the revolt's four leaders. By the end of 1692, most politicians elected by the rebel committee had been forced to resign and were replaced either by their predecessors or by their predecessors’ offspring (Christ, 1969; Schweizer, 1931; Ochs 1786). Because this local revolt was neither a precursor of class movements nor a conflict between guilds, this case offers a unique opportunity to analyze the relation between kinship ties and mobilization for revolts.

Our empirical analyses combine three unique datasets. The first dataset identifies the members of the city’s political elite during the revolt. The second dataset includes a list of the insurgents who mobilized for the revolt, extracted from a handwritten chronicle of the events. The third dataset documents parts of the genealogical database of the Historical Family Dictionary of Switzerland (hereafter HFLS) and contains genealogical information about 13,199 individuals living in Basel from 1150 to 1690. We establish a network of kinship relations of the 1793 politically relevant people living in Basel during the revolt that could have sided either with the elite or with the rebels (Geweke et al., 2022). The empirical findings indicate that kinship relations to the ruling elite are better predictors of an individual’s mobilization for revolts than kinship relations to the rebels.

This paper proceeds by presenting a private interest framework based on Tullock (1971) and by deriving the hypotheses in the second section. We then deliver an overview of the empirical case study. A fourth section presents the data and method used for the analyses, followed by the results and the discussion of our analyses.

## Kinship ties, private interests, and mobilization for revolts

We draw on Tullock’s (1971) formalization of the conditions under which a rational actor will join a revolution to derive our hypotheses. The equations are presented for purely illustrative purposes and are not intended to present a new formal model of mobilization for revolts.

Following Tullock’s example, we model an actor’s net expected payoffs from participating in a revolt, as in Olsson-Yaouzis, 2010, 286) and Marcum and Skarbek (2014, 239):

(1) $$U_{inactive}=A\cdot p+B\;\cdot \;\left(1-p\right)$$

(2) $$U_{mobilize}=A\;\cdot \;\left(p+p_{p}\right)+B\;\cdot \;\left(1-\left(p+p_{p}\right)\right)-C,$$

where *A* is the gross individual expected payoffs of mobilization for revolts in case of success. These payoffs refer to positions of power, financial resources, and social prestige: privileges that are awarded to mobilized actors after a successful revolt. *B* is the gross individual expected payoff in an unsuccessful revolt, defined as the privileges that actors maintain only if the old social order persists. The probability of success regardless of the rational actor’s contribution is *p*, and 1-*p* is the probability of failure. Rational actors believe that their own mobilization will increase the chances of the revolt’s success by *p*_*p*_. By mobilizing, they face the costs of mobilization for revolts *C*, which may include social ostracism and exclusion from social groups, as well as the risk of facing severe punishments such as imprisonment, torture, and execution if the revolt fails (Silver, 1974). The costs of mobilization for revolts are positive (*C* > 0) for all actors.

Tullock (1971) suggests that whenever enough actors are mobilized, an actor’s marginal contribution can be approximated to zero (*p*_*p*_≅0), because the presence of one additional person will not have a strong impact on the overall chances of success of the revolt. Thus, formula (2) becomes

(3) $$U_{mobilize}=A\cdot p+B\;\cdot \;\left(1-p\right)-C.$$

The private interest theory, as presented in equation (3), suggests that people are mobilized for revolts if *A* > *B*: if the gross expected individual payoffs in case of success exceed the gross expected individual payoffs in case of failure (Marcum and Skarbek, 2014, 239; Olsson-Yaouzis, 2010; Tullock, 1971, 286). With *C* > 0, the problem of free-riding arises: all actors face positive costs of mobilization and, thus, not mobilizing should be the rational choice. However, we posit that the costs of mobilization for revolts are not the same for all actors, which alters the framework especially for mobilization for smaller revolts. Even capital sentences could vary in their severity depending on the offense, with some death sentences being executed more cruelly than others. For instance, during the Middle Ages, execution by drowning was considered an honorable death, particularly for aristocrats, because it avoided spilling noble blood. Conversely, such serious offences as treason were punished with torture, public humiliation, and the harshest execution methods (Kesselring, 2003; Bellamy, 2004). In addition, the costs of mobilization for revolts also depend on an individual’s perception of the actual risk of being caught (Zhou and Wang, 2018). Finally, the degree of social ostracism may contribute to the variance of the costs of mobilization, as exclusion from social groups may have a greater impact on some individuals than others. By allowing the size of *C* to vary among individuals, we introduce a new condition for a rational actor’s mobilization for revolts, if *A* > *C*: if the gross expected individual payoffs exceed the expected individual costs of mobilization for revolts. In the following, we use this setting as a framework in which we posit that kinship relations affect the expected individual gross payoffs and costs of mobilization for revolts. Kinship relations can shift the rational calculations of individuals by increasing or decreasing their perceived gross payoffs and costs (Goldstone, 1994; Zhou and Wang, 2018). For example, the extent of social ostracism expected by actors might decrease if their kin also mobilize and, thus, their perceived costs of mobilization might decrease.

Research on altruism and social networks has shown that people are more supportive of close contacts such as family and friends than of occasional acquaintances and strangers (Stark, 1995; Schulze et al., 2001; Lubatkin et al., 2005). The frequency of communication, intimacy, and reciprocity, referred to as tie strength (Granovetter, 1973), influences the extent to which people support (Koster and Leckie, 2014; Verdery et al., 2012), reciprocate (Baldassarri, 2015; Baldassarri and Grossman, 2013), and cooperate with each other (Binzel and Fehr, 2009; Fowler and Christakis, 2010; Apicella et al., 2012; Melamed and Simpson, 2016). Within families too, altruism, cooperation and solidarity are unequally distributed and dependent on the distance between kin. Close kin are usually connected over low kinship distance by stronger ties than distant relatives with high kinship distance, because of their shared bloodline, their common upbringing, and stronger emotional bonds (Stark, 1995; Hicks, 1998). Accordingly, powerful people such as members of the ruling elite usually share their privileges with their close relatives and rarely have any interest in sharing them with their distant kin (Hicks, 1998; Schulze et al., 2001; Lubatkin et al., 2005). Increasing kinship distance is accompanied by decreases in altruism, solidarity, and cooperation.

Our framework considers two opposing groups of rational actors: the ruling elite and the rebels (Funcke and Franke, 2016; Olsson-Yaouzis, 2010). The ruling elite holds positions of power, substantial financial resources, and prestige. The rebels lack such privileges and aim to overthrow the old social order. Using Tullock’s (1971) model, we derive the expected net payoffs of mobilization for revolts for actors that are close or distant relatives of the elite or the rebels.^2^

### Close kin of the elite

Close kin of the elite usually profit from the privileged status of their family, either directly through nepotism, or indirectly through financial contributions, inheritances, and social status. They already have the privileges they would gain as mobilized actors in a successful revolt and thus have no incentives for disrupting the current social order. Therefore, their expected gross payoffs if the revolt succeeds are negligible (*A*≅0), and they expect positive gross payoffs if the revolt fails (*B* > 0) because this failure would preserve the favorable status quo and the privileges of the close kin of the elite. Conversely, if close kin of the elite mobilize for the revolt and thus antagonize their family, they can expect very harsh punishments, as they are likely to be tried as traitors rather than mere rebels. History provides numerous examples of close relatives of monarchs and rulers who were brutally executed after plotting against their own kin, such as Mary Queen of Scots, who was beheaded in 1587 after being convicted for treason against her cousin Queen Elizabeth I (Kesselring, 2003; Bellamy, 2004). Additionally, close kin of the elite would be the most affected by social ostracism and exclusion, as they would be deprived of family assets and stripped of their privileges irrespective of the outcome of the revolt. Although their well-connected positions in the network of kinship relations may potentially grant them strategic alliances and reduce their perceived risk of detection, overall, the costs of mobilization for revolts *C* for close kin of the elite can be estimated to be very high. Thus, for close kin of the elite, equation (3) becomes

(4) $$U_{mobilize}=B\;\cdot \;\left(1-p\right)-C,$$

with close kin of the elite having an interest in keeping the revolt small and its chances of success low because they expect high payoffs in case of failure of the revolt.

### Distant kin of the elite

Whereas the private interest of close kin of the elite are in preserving the status quo, the private interest assessment is different for distant kin of the elite. Because altruism mainly occurs among close kin, actors that are distantly related to the elite do not profit from their privileges. However, because they are still distantly related to the elite, the elite become an important reference group for social comparisons (Olson, 1965). For distant kin of the elite, the gross individual payoffs of mobilization in case of success are positive (*A* > 0), because a successful revolt would grant them the privileges they might feel entitled to by virtue of their noble origins (Hicks, 1998; Schulze et al., 2001). Above, we have seen that the gross individual payoffs in case of failure are the privileges resulting from the current social order. If distant kin of the elite are not advantaged from the current social order, for them the gross individual payoffs in case of failure are negligible (*B*≅0). The costs of mobilization for revolts for distant relatives of the elite are typically lower than those faced by close relatives of the elite. First, as distant relatives are often not considered family members, their participation in a revolt would be less likely to be considered treasonous (Hicks, 1998; Kesselring, 2003). Consequently, their punishments would likely be less cruel than those imposed on close kin. Second, their exclusion from family assets would have a lesser impact, as they are typically not included in such arrangements. Finally, their position in the network of kinship relations, while more peripheral than that of close kin of the elite, would still reduce the distant kin of the elite’s perceived risk of detection. For instance, in rare instances in premodern England, insurgent princes with distant relations to other powerful family dynasties were spared the death penalty by virtue of their kinship network (Hicks, 1998).^3^ Consequently, the perceived costs of mobilization for rational actors that are distant kin of the elite are lower than for rational actors that are close kin of the elite, and equation (3) becomes

(5) $$U_{mobilize}=A\cdot p-C$$

with

(6) $$C_{distant\;kin\;elite}<C_{close\;kin\;elite},$$

where mobilization for revolts is very attractive because the net expected payoffs of mobilization *U*_*mobilize*_ are positive only in a successful revolt. This argument is supported by (Gould, 1993; Kim and Bearman, 1997), who suggest that mobilization for revolts is particularly attractive to actors that have high incentives and face low risks coming from privileged network positions. From this, we derive our first hypothesis:

H1: Distant kin of the ruling elite are more likely to mobilize for revolts than close kin of the ruling elite.

### Close kin of the rebels

For close kin of the rebels, mobilization for revolts also yields positive gross individual payoffs only if the revolt succeeds (*A* > 0). In a successful revolt, the rebels might share their new power, financial gains, and social status with their close kin. Like distant kin of the elite, close kin of the rebels do not have any privileges to lose, which leads to negligible gross expected payoffs in case of failure (*B*≅0). However, the costs of mobilization are higher for close kin of the rebels than for distant kin of the elite. Close relatives of the rebels, who are not related to the elite, are unlikely to face charges of treason, and they will not be excluded from their family assets because their close kin also mobilize for the revolt. However, due to the absence of an advantaged position in the kinship network and of strategic alliances with powerful actors, they can expect higher risks of being detected and punished. Consequently, the net expected payoffs of mobilization for revolts for close kin of the rebels are determined by equation (5), but with

(7) $$C_{close\;kin\;rebels}>C_{distant\;kin\;elite}.$$

Thus, our second hypothesis can be derived:

H2: Distant kin of the ruling elite are more likely to mobilize for revolts than close kin of the rebels.

### Distant kin of the rebels

Finally, for distant kin of the rebels, mobilization for revolts is least attractive. For distant kin of the rebels, the gross individual payoffs of mobilization in case of success are approximately zero (*A*≅0). If the revolt succeeds the rebels are unlikely to share their hard-fought privileges with people that are only distantly related to them. Similarly, the gross individual payoffs in case of failure of the revolt are also negligible (*B*≅0), as distant kin of the rebels rarely have privileges to lose if the old social order is overthrown. However, their expected costs are the same as for close kin of the rebels because distant kin of the rebels also lack strategic alliances with powerful actors. This yields particularly unattractive net expected payoffs of mobilization for revolts:

(8) $$U_{mobilize}\;=-C,$$

where distant kin of the rebels undergo the risk of being punished without expecting any payoffs in return. Thus, our third hypothesis posits that

H3: Close kin of the rebels are more likely to mobilize for revolts than distant kin of the rebels.

We test these hypotheses by analyzing the impact of relations in a kinship network on the probability of mobilization for the *Einundneunziger Wesen*.

## Case study: The Basler revolt of 1691

The Basler revolt of 1691 provides an interesting case for studying mobilization for revolts. From 1529 to 1798, Basel was a city-state of the Swiss Confederacy, run by a complex system of political representation based on guild membership. Until the uprising of 1691, the city’s highest authority was a parliament chamber that included two mayors and two vice-mayors (Christ, 1969; Degen et al., 2017).^4^ However, parliamentarians could hold their positions for life, and resignations were rare. By the end of the 16th century, a small number of large and influential families had established an effective oligarchy over the city (Burghartz, 1993; Christ, 1969; Degen et al., 2017; Kutter, 1991). Further, throughout the second half of the 17th century, Basel was constantly confronted with diplomatic tensions with neighboring France, and some even accused the two mayors of the city of having been corrupted by French king Louis XIV. Among the accusers was Jakob Henric-Petri, a distant relative of the two mayors and holder of a marginal parliamentary position. On the night of January 26, 1691, he summoned other concerned citizens and created a rebel committee (Schweizer, 1931). Membership of the rebel committee was restricted to male citizens with an occupation registered in one of the 15 guilds of the city, thus excluding women, peasants, and other underprivileged citizens (Ochs, 1786; Schweizer, 1931; Burghartz, 1993). Almost every mobilization was enacted by the members of the rebel committee alone, and spontaneous mobilizations of other insurgent citizens are not documented in the historical accounts (Ochs, 1786; Schweizer, 1931; Universitätsbibliothek, 17th/18th century). For these reasons, we consider only the members of the rebel committee as rebels.

On March 24, 1691, the rebels laid siege to the front of the city hall and forced numerous allegedly corrupted parliamentarians to resign (Christ, 1969; Degen et al., 2017; Ochs, 1786; Schweizer, 1931). The vacant positions were then assigned to people elected by the rebel committee. However, the rebels’ success was short-lived, and the elite regained control of the parliament and city a few months later. Three leaders of the revolt were publicly executed; the other rebels were punished with monetary fines and bans from churches, pubs, and taverns (Schweizer, 1931; Ochs, 1786). By the end of 1692, the old social order had been re-established, and the powerful families managed either to reinstate the parliamentarians that had lost their positions a few months earlier or to assign the vacant positions to younger members of the parliamentarians’ families (Christ, 1969; Schweizer, 1931; Ochs, 1786).

The historical context in which the *Einundneunziger Wesen* occurred does not fit the main historical narratives of early modern Europe (Geweke and Rost, 2023). First, Basel was a merchant city with high trade volumes and commercial success (Röthlin, 1986). As a member of the Swiss Confederacy, Basel was not involved in the major military conflicts of the time and was not governed by an absolutist ruler (Geweke and Rost, 2023). On the contrary, Basel maintained a high level of parliamentary activity throughout the early modern period (Christ, 1969; Degen et al., 2017). The revolt of 1691 thus did not aim to overthrow an absolutist ruler, nor was it triggered by ongoing military conflicts as were many other social uprisings (Skocpol, 1979; Zhang, 2021). Instead, the revolt targeted the ruling oligarchy and was initiated from within the city’s social and political elite (Doehne et al., 2023).

Second, the revolt of 1691 was not a bottom-up social movement, like the Swiss peasant uprisings of 1653 (Ochs, 1786) or the larger mass revolutions that would coincide with the Swiss Confederacy by 1798 (Degen et al., 2017; Ochs, 1786). The revolt of 1691 was a purely urban, well-organized uprising in which citizens willing to mobilize had to enroll in a structured, official rebel committee. Thus, in contrast to class-based revolts that can be explained by discontent among an underprivileged agrarian class vis-à-vis a landed elite (Nieva, 2021), the revolt of 1691 played out among the urban citizenry and therefore does not meet the criteria of a class movement. Moreover, because the rebel committee included members of all 15 guilds of the city, we can dismiss any interpretation of the revolt of 1691 as a conflict between guilds.

These characteristics of the *Einundneunziger Wesen* allow us to focus on the impact of kinship relations on mobilization for revolts*.* Historical accounts have also underlined the importance of kinship relations for a thorough understanding of this revolt. Historians have suggested that the revolt of 1691 was an escalation of conflicts between rival families (Ochs, 1786; Kutter, 1991). However, many families were represented on both sides of the revolt, suggesting that the dynamics of mobilization were more fine-grained. We contest this historical interpretation and suggest that the revolt evolved around distant relatives of the Basler elite, who mobilized to change the status quo and gain access to power.

**Figure 1.**
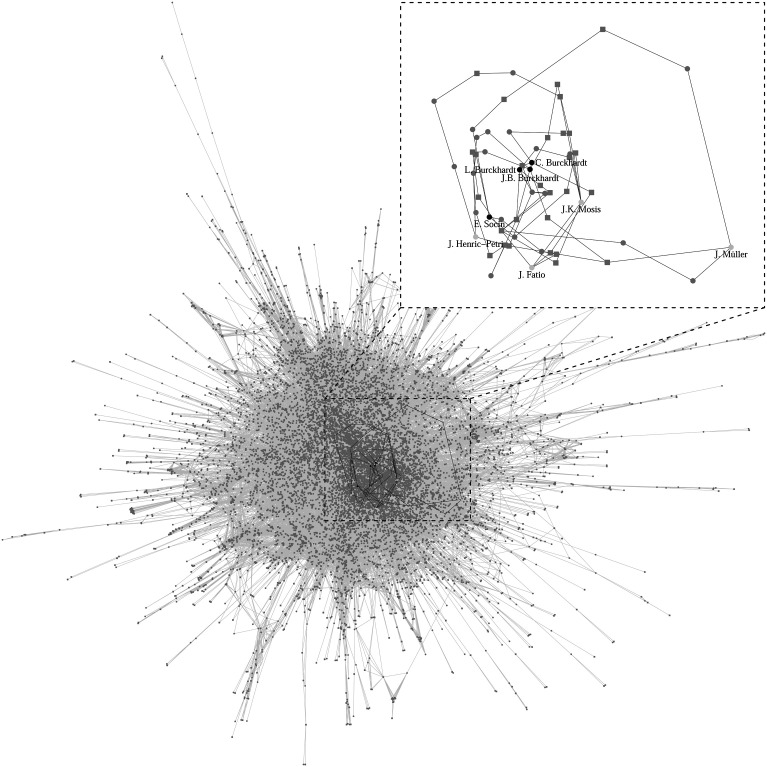
Graphical representation of the kinship network of the rebel leaders and the most influential politicians of 1691. Note: Edges represent spousal, first-, second, or third-degree kinship relations between nodes (i.e., parent and sibling). Males are denoted by circles, females by squares. Light-gray nodes identify the four leaders of the rebels, black nodes the four major politicians. Gray nodes identify other citizens of Basel. The subplot shows the shortest paths connecting the four rebel leaders and the four politicians.

This pattern clearly emerges when the four leaders of the rebels are considered. Henric-Petri openly denounced his distant kin and enlisted rebels from the guilds (Ochs, 1786; Schweizer, 1931). Among them were Johannes Fatio, Konrad Mosis, and Johannes Müller, who later took over Henric-Petri’s role as revolt leaders.^5^ Figure 1 shows that all four leaders of the revolt were distant relatives of the two most important families in the city, the Burckhardts and the Socins, who by 1690 monopolized the four highest offices of the parliament. In his memoirs, Henric-Petri, the only leader of the rebels who managed to escape the city and the death penalty, directly attacks the two families, accusing them of being corrupt, dishonest, and of unjustly living off the city’s resources:

Then came the nearest cousin of the Socin family, whose father had also lived off the public-good [of Basel] for over 200 years, especially from the resources of the St. Jakob parish, where his brother Emanuel, the mayor, and Abel, the director of the parish, had recommended him …. Many Burckhardts, or rather most of them, followed the example of the Socin family … and accommodated family members to clerical positions where they could live off the rich donations to the parishes and steal from the common good. (Henric-Petri 1693, 23; authors’ translation)

Later, he states that he would have been a more suitable candidate for the role of mayor of the city, because “neither me nor my ancestors, who have had numerous political positions over the last 200 years, have ever enjoyed privileges to the extent [of the Burckhart and Socin] and have never manifested interest in becoming wealthy from the city of Basel” (Henric-Petri 1693, 24; authors' translation).^6^ Although we cannot confirm whether Henric-Petri’s allegations on the honesty of his kin were legitimate, this quotation underscores the importance of kinship relations in the Basler society of the early modern period. Henric-Petri used his descendance to legitimize his own rights to higher parliamentary positions.

Henric-Petri was the only leader of the rebels who had the opportunity to write his testimony of the revolt. However, the handwritten chronicles of the revolt of the *Einundneunziger Wesen* report extracts of parliamentary debates and public speeches held by the rebels. In several of these extracts, Johannes Fatio, who was also related to the Burckhardt family, referred to the Burckhardt’s nepotistic practices as the “city’s disgrace” and invoked a “divine punishment” upon the corrupt family (Universitätsbibliothek 17th/18th century, 412–13; authors’ translation). Thus, there is anecdotal evidence in favor of our hypothesis that distant kin of the ruling elite were those who first mobilized for the revolt.

## Data and empirical strategy

### Data

Figure 1 illustrates the kinship ties between important actors for the revolt in a network of kinship relations. We derived this network from the HFLS genealogical database, which contains genealogical information on 13,199 individuals living in Basel from 1150 to 1690.^7^ This data was gathered from family trees stored in multiple archives and from the personal research of private genealogists (HFLS 2017; Lutz 1819; Stroux 2012; Geweke et al., 2022) and thus varies in depth and completeness; some smaller families have cropped family trees with dead ends and imprecise information. Thus, we decided to restrict our analyses to the largest connected component of the network, termed the giant component, which includes 12,217 nodes and 80,568 edges connecting every person in the giant component to their parents, children, and siblings. Our framework proceeds from the intuition that increasing kinship distance is accompanied by a decrease in the strength and intensity of altruism, solidarity, and cooperation. Our research thus pivots on the analysis of more complex kinship relations that connect between nuclear families.

**Figure 2.**
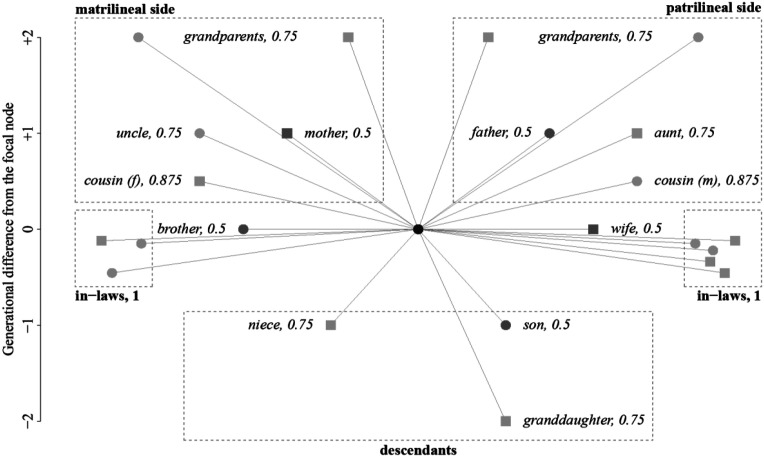
Kinship distance and extended family ties; an example of an ego network. Note: Males are denoted by circles, females by squares. The focal node in the ego network is colored in black; dark-gray nodes identify close kin: parents, siblings, and children. Light-gray nodes identify extended kin, such as grandparents, grandchildren, cousins, and in-laws. The number next to the kinship relation denotes the weight of the edge connecting ego to each alter, which corresponds to the kinship distance between ego and alter. All in-laws have the same weight of 1, as they do not share the same bloodline. The dashed boxes exemplify the four different types of relation.

From the HFLS genealogical data, we derived additional kinship ties by relying on the socio-anthropological understanding of kinship systems (Parsons 1943). For example, ties connecting males to their parents’ brothers correspond to relations between uncles and nephews and ties connecting males their sisters’ husbands or to their wives’ brothers define relations between brother in-laws. Figure 2 shows how the network is extended by introducing the additional kinship ties. This exemplary representation of an ego network shows the various weights of the edges connecting an actor here termed ego, to other actors termed alteri, and the labels that these relations have in our measure. This process does not add new nodes to the network, but it increases the number of edges connecting existing nodes. Accordingly, the final network has the same number of nodes (12,127) but a higher number of edges (311,047).

Within the network, we manually identified members of the ruling elite and individuals that mobilized for the revolt. The information that allowed us to identify these actors, such as their names and surnames, birth and death years, and guild memberships was gathered from two original manuscripts dating from the 17th and 18th centuries. The first manuscript is an extract from the official register of political positions of Basel until 1798. We defined the ruling elite as 50 politicians that held office in the most powerful parliament chamber of the city by 1690 (Linder 1748–1796). The two mayors and vice-mayors of the city are included. The second manuscript is a list of the 92 rebels that participated in the revolt, extracted from the official chronicle of the revolt (Universitätsbibliothek 17th/18th century).

### Measures

Our main dependent outcome measure is a binary variable assessing whether actors mobilized for the revolt. To participate in political life, even as a protester, a Basler had to be male, older than 15, and enrolled in a guild. Thus, our analyses focus on the 1793 people that fulfilled these criteria in 1690, as other individuals would not be allowed into the rebel committee.

**Figure 3.**
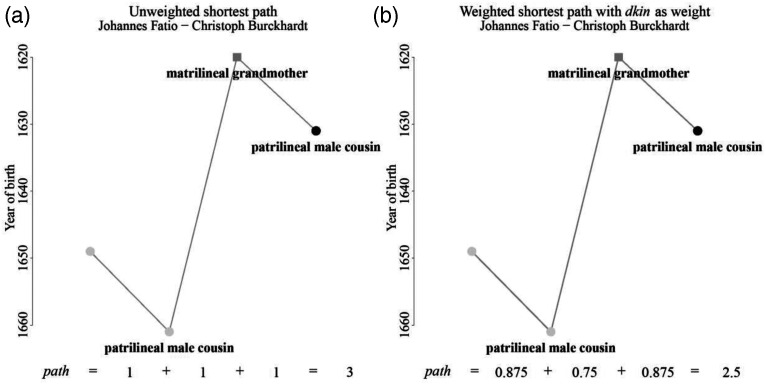
Operationalization of kinship distance. Note: Males are denoted by circles, females by squares. Light-gray nodes identify rebels, black nodes identify elite members, gray nodes identify nonmobilized individuals. In Figure 3(A), the value assigned to the path corresponds to the length of the shortest path. In Figure 3(B), the value of the path corresponds to the kinship distance, which is the sum of the weights of the shortest path connecting two individuals.

To operationalize the kinship distance, we weighted the edges between an ego and its related alteri by a coefficient of relatedness (Koster and Leckie, 2014; Wright, 1922). This measure assesses the proximity of two related people according to their shared bloodline. For example, parents and their children have on average 50% of the same blood; grandparents and grandchildren only 25%. The coefficient of relatedness accounts for these differences in shared bloodlines and assigns each relation between people a score between 0 and 0.5. The score 0 means that two people are not blood-related at all, whereas 0.5 represents the maximal relatedness between two people, as in the case of parents, children, and siblings. We inverted the coefficient of relatedness with the formula displayed in equation (9) and labelled the resulting measure kinship distance

(9) $${dkin}_{ego,alter}=1-{coeff.rel.}_{ego,alter},$$

where $coeff.rel._{ego,alter}$ is the coefficient of relatedness of the edge connecting an ego to an alter. In our new measure, $dkin_{ego,alter}$ , 0.5 represents the smallest possible kinship distance between two people, and one represents non-blood-related people. Figure 2 identifies distances that are associated with various kinship relations by this measure. This operationalization allows measurement of the kinship distance between people that are directly related to each other. To quantify the kinship distance between an ego and an alter that is only indirectly related to ego, such as ego’s brother-in-law’s cousin, we identified the shortest path between the two nodes using the Dijkstra algorithm for weighted networks. The resulting score is the sum of the weights of all the edges on the shortest path between ego and alter:

(10) $${dkin}_{ego,alter}=\overset{n-1}{\underset{i=1}{\sum }}\overset{n}{\underset{j=1}{\sum }}{dkin}_{i,j},$$

where *n* represents the number of nodes on the shortest path between ego and alter, $dkin_{i,j}$ the kinship distance assigned to each edge indexed, with *i* the node from which the edge starts and *j* the node with which the edge ends. Figure 3 shows a graphical representation of one shortest path connecting two indirectly related individuals: Johannes Fatio, one of the rebels, and Christoph Burckhardt, one of the elite. In Figure 3(A), the value of the unweighted shortest path corresponds to the number of edges on the path, while in Figure 3(B) the value of the weighted shortest path is given by the sum of the kinship distances of all the edges on the path. This comparison illustrates two advantages of using the weighted measure of kinship distance. First, the weighted network assesses the proximity of two related people according to their shared bloodline and thus accounts for our theoretical assumption that altruism, cooperation, and solidarity are unequally distributed in families and dependent on the kinship distance between relatives. For example, in Figure 3(a), the patrilineal male cousin of Johannes Fatio could be replaced by a closer relative, such as Fatio’s father, without this having any effect on the final value of the shortest path. The unweighted shortest path only considers the number of edges, which would remain the same, 3. In Figure 3(b), however, this substitution would have an impact on the final value of the weighted shortest path, as the weight of fathers, 0.5, is lower than the weight of cousins, 0.875. The new kinship distance, 2.125, would thus be smaller than the original, 2.5. Second, our approach helps assess kinship relations that exist but do not have official labels in the traditional socio-anthropological kinship systems, such as ego’s cousin’s grandmother’s cousin. Our operationalization of kinship distance offers a means of describing such unlabeled relations with numbers and allows comparisons between relations. For example, the kinship distance between an ego’s cousin’s grandmother’s cousin is 2.5, which is smaller than the distance between an ego and ego’s father’s grandmother’s cousin: 2.125.

Using the approach described above and represented in Figure 3(b), we calculate the kinship distance between all 1793 male Baslers that could potentially have become rebels in 1690, which results in a 1793 × 1793 matrix with the kinship distances as entries. Without the diagonal, which pairs each node to itself, the resulting dataset includes 3,213,056 dyads.

We include numerous control variables to check whether potential confounders influenced an ego’s mobilization for the revolt. At the individual level, we control whether the revolt was an intergenerational conflict by including the age of ego in 1690, when the revolt started. We also control for ego’s occupation and geographical origins.

Further, we introduce a categorical variable at the dyadic level that indicates the family relationship between an ego and an alter. We construct this variable by extracting the label of the first edge on the shortest path and then categorizing it into one of four groups (also visible in Figure 2): in-laws, descendants, matrilineal side, and patrilineal side. Thus, if the first edge on the shortest path connecting ego to alter is an edge connecting ego to ego’s patrilineal cousin, this connects ego and alter via members of the patrilineal side of ego’s family. If the first edge on the shortest path is an edge connecting ego to ego’s brother in-law, either ego’s wife’s brother or ego’s sister’s husband, this connects ego and alter via in-laws. This variable enables us to control whether some specific types of relations were more likely to lead to mobilization than others.

Table 1 presents descriptive statistics of all variables and reports the number of dyadic and individual observations. We observe 3,213,056 dyadic relationships between 1793 people, and 92 of these 1793 people were identified as rebels.

**Table 1.** Summary statistics.

Variable	Dyadic obs	Individual obs	Mean	SD	Min	Median	Max
Role of alter	3,213,056	1793					
Elite Alter	89,600	50					
Rebel Alter	164,864	92					
Neutral Alter	2,958,592	1651					
Kinship Distance	3,213,056		3.59	1.03	0.50	3.50	10.50
*Log* (kinship distance)	3,213,056		1.24	0.31	−0.69	1.25	2.35
Age Of ego	3,213,056	1793	35.13	14.41	9.00	33.00	95.00
*Log* (Age of ego)	3,213,056	1793	3.47	0.42	2.20	3.50	4.55
Side Of relation ego–alter	3,213,056						
Matrilineal	278,284						
Descendants	1,205,532						
In laws	1,342,614						
Patrilineal	386,662						
Occupation Of ego	3,213,056	1793					
State clerk	21,504	12					
State politician	86,016	48					
Artist	23,296	13					
Cleric	132,608	74					
Craftsman	2,152,192	1201					
Educator	100,352	56					
Healer	193,536	108					
Host	75,264	42					
Lawyer	53,760	30					
Military man	69,888	39					
Trader	301,056	168					
Unknown Occupation	3584	2					
Origins Of ego	3,213,056	1793					
Abroad	202,496	113					
Basel county	35,840	20					
Basel city	2,956,800	1650					
Switzerland	17,920	10					
Degree centrality of alter	3,213,056	1793	52.43	41.92	2	44.00	360
Avg. kinship distance from elite		1793	3.29	0.64	2.32	3.14	6.64
Avg. kinship distance from rebels		1793	3.60	0.62	2.72	3.42	7.05

### Method

Our main dependent variable is binary, with one indicating that ego mobilized for the revolt and 0 indicating no mobilization. We test our hypotheses by running dyadic logistic regression models with clustered standard errors for the 1793 egos influencing mobilization against the elite in ego–alter dyads. Five occupational groups—artists, clerics, educators, state clerks, and military men—predict failure perfectly, meaning that no actor belonging to these occupational groups mobilized for the revolt. To deal with the issue of complete separation, and as robustness checks for the dyadic analyses, we compute traditional logistic and Firth logistic-regression models at the individual level, where the actors’ probability of mobilization is modeled on their average kinship distance from the rebels and from the ruling elite. The Firth regression model is a classification model that calculates the estimates with penalized maximum likelihood. It has been developed to calculate coherent and unbiased models in the presence of small, imbalanced, and separated datasets (Firth 1993). We computed the Firth logistic regressions only at the individual level, because Firth models can only be specified for small samples. The penalized maximum likelihood estimation used in these models is complex and exceeds the computational capacity of most machines if run on large-scale datasets such as our dyadic dataset, which contains over three million observations.

## Results

Table 2 provides an overview of the network of kinship relations that underlies our analyses. The average degree of the members of the ruling elite, the number of direct kinship relations counted in the network of Basel citizens, is higher than the average degrees of both the rebels and neutral individuals. This result is plausible: recall that the network is derived from the genealogical data of Swiss families; thus, it is not surprising that elite members had larger families and better documented family trees than less privileged individuals. Moreover, in the 17th century, citizen rights were given especially to family members of the elite, which would explain why less privileged people are less represented in the genealogical dataset. Examining the average kinship distance by dyad types shows that elite members were more closely related to other elite members than to the rebels or to neutral individuals. Interestingly, the rebels were not as closely related to each other. This can be seen as a first indication that kinship relations among rebels do not seem to have played a role in mobilizing them.

**Table 2.** Summary of network data.

Edges	311,047
Nodes	12,127
citizens of Basel in 1691	4406
Of which male citizens aged 15 or older	1793
Of which rebels	92
Of which elite members	50
Of which neutral	1651
Average degree	52.43
Rebels	52.41
Elite	88.84
Neutral	51.32
Diameter	11.50
Average kinship distance in dyadic dataset	3.59
Rebels–rebels	3.60
Rebels–elite	3.33
Rebels–neutral	3.61
Elite–elite	2.97
Elite–neutral	3.30
Neutral–neutral	3.61

### Dyadic-level analyses

**Figure 4.**
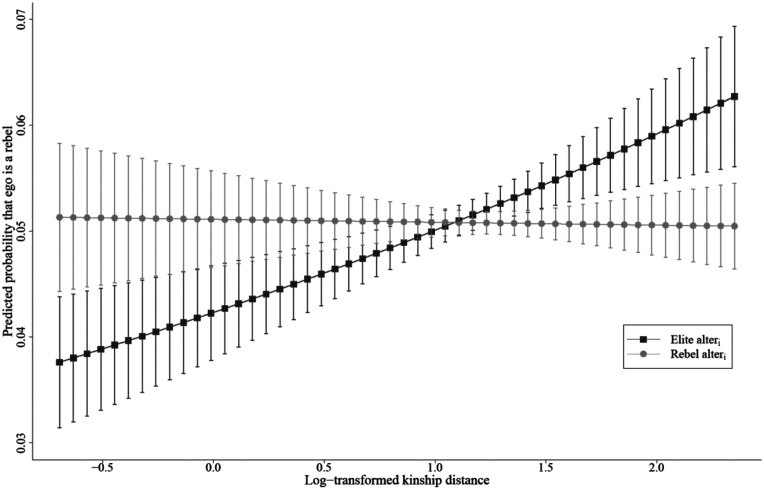
Effect of kinship distance on the probability that ego is a rebel; dyadic level. Note: The effects displayed in this figure refer to Model one in Table 3. The bars represent the 95% confidence intervals.

Table 3 documents the dyadic analyses of ego’s likelihood of mobilization for the revolt, dependent on the kinship distance between an ego and alteri who had various roles in the revolt. Figure 4 illustrates the predicted marginal effects of the results in Model 1.

**Table 3.** Dyadic logistic regression models on the probability that ego is a rebel.

	Logit models			
	(1)		(2)	
	B	SE	B	SE
Role of alter (ref.: Elite member)				
Rebel	0.198**	(0.072)	0.141†	(0.073)
Neutral	0.145**	(0.054)	0.111*	(0.056)
*Log* (kinship distance)	0.177***	(0.044)	0.098*	(0.046)
Rebel Alter × *log* (kinship distance)	−0.182**	(0.058)	−0.134*	(0.059)
Neutral Alter × *log* (kinship distance)	−0.130**	(0.045)	−0.098*	(0.046)
Side of relation (ref.: in laws)				
Patrilineal			−0.138***	(0.008)
Matrilineal			−0.023*	(0.009)
Descendants			−0.370***	(0.006)
*Log* (Age of ego)			1.235***	(0.007)
Occupation of ego (ref.: craftsman)				
Healer			0.659***	(0.008)
Host			0.534***	(0.013)
Lawyer			−0.736***	(0.025)
State politician			−1.334***	(0.024)
Trader			−0.164***	(0.009)
Artist			−15.654***	(0.008)
Cleric			−15.891***	(0.005)
Educator			−15.855***	(0.005)
State clerk			−15.783***	(0.008)
Military man			−15.736***	(0.006)
Unknown Occupation			−16.004***	(0.019)
Origins of ego (ref.: Basel city)				
Abroad			−0.052***	(0.010)
Basel county			−0.296***	(0.026)
Switzerland			−15.558***	(0.011)
Constant	−3.119***	(0.053)	−7.165***	(0.061)
Observations	3,213,056		3,213,056	
clusters	1793		1793	

^†^ *p* ≤ .10, * *p* ≤ .05, ** *p* ≤ .01, *** *p* ≤ .001.

Hypothesis 1 predicted that distant kin of the ruling elite could expect a higher utility for mobilization for the revolt than close kin of the ruling elite. Our analyses support this claim. Close kin of the elite were markedly less likely to participate in the revolt than distant kin (see Model one and Figure 4). The interaction term in Model one shows that a 10-fold increase in kinship distance from the elite increases the odds of ego mobilizing for the revolt by 19.3% ( exp(0.177)=1.193) . However, the predicted probability of ego mobilizing for the revolt is comparatively small. This can be derived from the marginal effects of kinship distance in Figure 4, where the highest slope estimate is 6.2%. This small effect size is partly due to the very low incidence of rebels in the network (92 rebels and 1701 nonrebels), which results in a similarly low incidence of rebel dyads in the dyadic dataset (164,864 rebel dyads and over three million nonrebel dyads). Nevertheless, the effect is observable and statistically significant and holds when the control variables are added to the model (see Model 2).

Hypothesis 2 predicted that distant kin of the ruling elite can expect a higher utility for mobilization for revolts than close kin. The findings in the kinship network support this hypothesis. This effect can be clearly seen in Figure 4, which graphically represents the marginal effects of kinship distance to alteri with different roles: either members of the elite or rebels. The slopes show that the probability of ego being a rebel decreases with increasing kinship distance from the rebels, whereas it increases with increasing kinship distance from the elite. Hypothesis two can thus be tested by comparing the two highest extremes of the estimated slopes, which represent close kin of the rebels and distant kin of the elite. For example, the closest relative of a rebel (that is with $dkin_{ego,rebel}=0.5$ ) has a predicted probability of mobilization of 5.1%, whereas the predicted probability of mobilization for the most distant relative of an elite member (that is $dkin_{ego,elite}=10.5$ ) is 6.2%. This difference of 1.1 percentage points is rather small and again suggests that the effects are comparatively small. Nevertheless, as Figure 4 shows, this effect is clearly observable and statistically significant. This is confirmed by contrasting the average marginal effects of dyads connecting ego to the rebels and of dyads connecting ego to elite members (Χ=11.62,p<0.01) . Moreover, this result does not change after the introduction of control variables in Model 2.

Finally, Hypothesis 3 predicted that close kin of the rebels can expect a higher utility for mobilization for revolts than distant kin of the rebels. The analysis does not support this hypothesis. The effect of kinship distance from rebel alteri on the mobilization probability of ego is effectively negligible (see Model one and Figure 6). A 10-fold increase in the kinship distance from rebel alteri decreases the odds of ego being a rebel by merely 0.5% ( exp(−0.005)=0.995) . This effect is negligible, and we can thus assume that the kinship distance from rebel alteri did not influence mobilization for the revolt. We must therefore reject Hypothesis 3: close kin of rebels are not more likely to mobilize for revolts than distant kin of rebels.

The control variables introduced in Model two show plausible and consistent coefficients. The types of kinship relations connecting egos to alteri indicate that patrilineal kinship relations, matrilineal kinship relations, and relations from the descendants’ side were less likely to foster mobilization for the revolt than edges that connect egos to their in-laws. Older individuals were more likely to become rebels, suggesting that the revolt of 1691 was enacted by individuals that had already reached an age that awarded them eligibility in Basler politics. Furthermore, healers and tavern hosts were more likely to mobilize for the revolt than craftsmen, whereas lawyers, state politicians, and traders were less likely to mobilize than craftsmen.^8^ Finally, people from outside and thus with fewer relationships to the ruling elite were less likely to mobilize for the revolt than people with origins in Basel city.^9^

### Individual-level analyses

**Figure 5.**
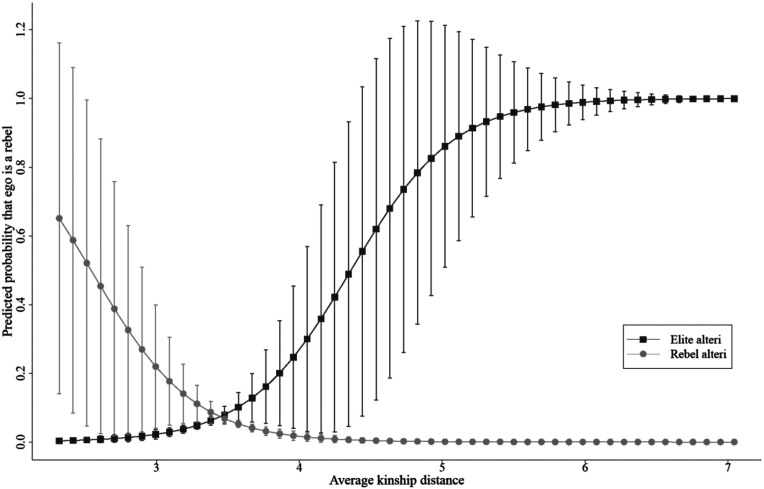
Effect of kinship distance on the probability that ego is a rebel; individual level. Note: The effects displayed in this figure refer to Model one in Table 4. The bars represent the 95% confidence intervals. The logistic regression in Model one is calculated from individual-level data, and due to the underlying distribution of observations, the resulting curves are s-shaped.

Because the decision to mobilize is ultimately an individual choice, we also calculated individual-level models as a robustness check for the results of our dyadic approach. Our original variable of interest, the interaction term between kinship distance and the role of alteri, only works in the dyadic analysis. For the analyses of the individual’s choice, we computed logistic regression models with average kinship distance from members of the ruling elite and average kinship distance from rebels as explanatory variables. To deal with the issue of complete separation discussed in the Method section, we calculate Firth logistic-regression models. The results shown in Table 4 and illustrated in Figure 5 indicate the marginal effects of the average kinship distance from rebel and elite alteri. Across all models, the effect of the average distance from elite members is significant, positive, and similar to that observed in the dyadic analysis. This supports the finding of the dyad model and Hypothesis 1, which posited that close kin of an elite are less likely to mobilize for a revolt than distant kin of the elite. As can be observed in Figure 5, the predicted probabilities are higher than in Figure 3, indicating that the effect sizes in the individual model are larger than in the dyadic model. This is probably due to the variable of interest being the average distance from all the rebels or elite members rather than the distance from one alter in a specific dyad. Figure 5 also supports Hypothesis 2, showing that distant kin of the ruling elite were significantly more likely to mobilize for the revolt than close kin of the rebels. The marginal effects of the individual report depict larger effect sizes and show that the predicted probability of distant relatives of the elite is almost 100%, whereas close relatives of the rebels have a lower predicted probability of mobilization for revolts of around 65%. However, in Figure 5, the confidence intervals are very large for close kin of the rebels $\left(avg.dkin_{ego,elite}\leq 3\right)$ , partly because few observations are available within this range. For the higher values of kinship distance, the confidence intervals are smaller, and the observed trends confirm Hypothesis 2. Finally, in contrast to the findings in the dyadic analysis, the individual level results also support Hypothesis 3. The effect of the average distance from the rebels is significant, negative, and comparatively large, as suggested by the fall in the grey slope in Figure 5. This suggests that close kin of the rebels were more likely to mobilize for the revolt than were their distant kin.

**Table 4.** Individual logistic regression models on the probability that ego is a rebel.

	Logit models				Firth logistic-regression models			
	(1)		(2)		(3)		(4)	
	B	SE	B	SE	B	SE	B	SE
avg. kinship distance to elite members	2.771**	(0.892)	2.212*	(0.946)	2.747**	(0.876)	2.183*	(0.886)
avg. kinship distance to rebels	−2.804**	(0.931)	−2.233*	(0.954)	−2.766**	(0.907)	−2.190*	(0.902)
*log* (age of ego)			1.304***	(0.285)			0.737***	(1.850)
Occupation of ego (ref.: craftsman)								
healer			0.708^†^	(0.369)			0.731*	(0.340)
host			0.519	(0.556)			0.614	(0.519)
lawyer			−0.553	(1.082)			−0.172	(0.854)
state politician			−1.163	(1.058)			−0.771	(0.843)
trader			−0.021	(0.406)			0.026	(0.375)
artist			−15.515***	(0.405)			−0.353	(1.373)
cleric			−15.721***	(0.257)			−2.219*	(1.386)
educator			−15.637***	(0.253)			−1.849^†^	(1.415)
state clerk			−15.550***	(0.408)			−0.258	(1.449)
military man			−15.563***	(0.270)			−1.424	(1.421)
unknown Occupation			−15.915***	(1.970)			1.016	(1.571)
Origins of ego (ref.: Basel city)								
abroad			0.037	(0.413)			0.077	(0.388)
Basel county			−0.189	(1.173)			0.159	(0.859)
Switzerland			−15.471***	(0.559)			−0.191	(1.394)
constant	−1.987**	(0.744)	−6.762***	(1.296)	−2.029**	(0.713)	−6.725***	(1.252)
observations	1793		1793		1793		1793	

† *p* ≤ .10, * *p* ≤ .05, ** *p* ≤ .01, *** *p* ≤ .001.

## Discussion

In this study, we have developed a new perspective on the relation between pre-existing networks and mobilization for revolts. We have proposed a simple framework that integrates social networks into rational assessments of the payoffs and costs of mobilization, and we offer a preliminary microfoundational explanation for the mechanisms that underlie mobilization for revolts. We tested our framework by analyzing the well-documented historical case of Basel’s revolt of 1691. Our findings show that the strength of kinship relations to the city’s ruling elite affected a person’s probability of mobilization for revolts. We find that actors with distant kinship relations to the ruling elite were the most likely to mobilize against their powerful relatives. Distant kin of the elite were even more likely to mobilize for the revolt of 1691 than close kin of the rebels. This result expands on findings in previous literature, which identified close social relations with other activists as the main predictor of mobilization.

Our study shows that a relational perspective can enrich rational understanding of mobilization for revolts. The presented historical case study suggests a new, intriguing way of thinking about revolts that develop in social networks polarized by the presence of mobilized individuals, the rebels, and of actors opposing the mobilization effort, the elite. By virtue of their position in the network of kinship relations, distant relatives of the elite expect high payoffs of mobilization for revolts yet face the lowest costs of mobilization for revolts. Thus, rational actors that are distant kin of the elite are the most likely to mobilize against the elite, even more than close kin of the rebels. Our framework reinforces the intuition of Roger Gould’s pioneering work on the Whiskey rebellion in Pennsylvania in 1794 (Gould 1996), which is that disadvantaged members of the elite are more likely to revolt than those who are closely connected to already mobilized actors.

History offers many examples of neglected clans of powerful families mobilizing against their privileged relatives, especially in nondemocratic regimes, where positions of power are more likely to be awarded within traditional kinship structures. Consider, for example, the English Wars of the Roses in the 15th century, where rival branches of families with royal descent mobilized against each other to produce the longest civil war in the history of England (Hicks 2014). However, a basic prediction of the private interest framework is that distant kinship relations to the elite may explain mobilization in contemporary settings as well. On a topical note, we speculate that this framework may also generalize to nonviolent conflicts within family dynasties. For example, the 200-member Swarovski family clan has been subject to disputes about the leadership of the Swarovski firm. In recent years, peripheral members of the family have fought for representation in the company’s board. Protracted legal disputes between opposing family factions ultimately led to a leadership change and to the appointment of the first external CEO in Swarovski’s business history.

Against this backdrop, our proposed framework, combined with new forms of data from social media and automated document analysis, may offer new opportunities to study the importance of kinship structures for the outbreak of revolts, or even conflicts generally, in contemporary settings. We show that strong relations, such as those formed in kinship networks, can have a diverging effect rather than a bridging one and can lead actors to engage in conflicts with their own kin. In fact, our analyses suggest that in a mobilization for revolts, distant kinship relations to the targets of the revolt, the elite, are more important predictors of mobilization than close kinship relations to already mobilized individuals, the rebels.

## Notes

1. These are not mutually exclusive categories, and people can be related to both the rebels and the elite. Our network of kinship relations accounts for this possibility, and our focus on dyads in the main analyses rather than on individuals (see Data and empirical strategy) allows us to analyze the impact of single kinship ties to elite members or rebels without any simplification or data loss.

2. As in Tullock (1971), our framework conceives only two alternatives for rational actors: to mobilize or not to mobilize for revolts. An in-depth study of the preconditions of mobilization, like coalition formation (Nieva, 2021), or of intermediate forms of mobilization prior to the revolt itself (Funcke and Franke, 2016), would go beyond the scope of this paper.

3. For example, during the War of the Roses in 1413, Henry Percy, Duke of Northumberland, tried to overthrow the newly crowned English King Henry V and was arrested. The king spared Henry Percy punishment and restored his title of Duke of Northumberland because of his kinship connections to the noble families of northern England (Hicks, 1998, 2014). Hicks also lists other meaningful examples of rebels whose titles were restored by virtue of their kinship ties during the conflict.

4. Basel was not a republic. Only people living in the city with possessions and occupations had political rights. Peasants living in the countryside, street beggars, and women were excluded (Christ, 1969; Degen et al., 2017).

5. Henric-Petri was demoted after he decided to stand as candidate for a second parliamentarian position that had become vacant during the revolt. In 1692, he managed to escape Basel and the death penalty, seeking asylum in various German cities. He died in Vienna, probably in 1695 (Schweizer, 1931; Ochs, 1786).

6. According to official register of political positions of Basel, by 1690 the Henric-Petri family had been represented in the parliament since 1554, albeit not continuously (Linder 1748–1796). The estimate provided in the quotation (“over 200 years”) is thus probably incorrect, although the official transcripts for the early 16th century were incomplete. Only one member of the Henric-Petri family held a position in the highest parliament chamber for more than 1 year: Sebastian Henric-Petri (1546–1627), who was a member of the “secret council”, the chamber responsible for domestic and foreign policy decisions (Geweke and Rost, 2023), from 1609 to 1627.

7. We excluded people born after 1690 from our sample to avoid reverse causality.

8. Note that artists, clerics, educators, state clerks, military men, and two individuals of unknown occupation predict failure perfectly, hence the strong negative coefficients. To deal with the issue of complete separation in our data, we have performed robustness checks with Firth logistic regressions at the individual level (see Table 4), which confirm the insights of the main analyses. Firth logistic regressions at the dyadic level were not possible because of the size of the dyadic dataset.

9. With this variable, the issue of complete separation also arises. Our robustness checks in Table 4 again confirm the insights of the main model.
